# Treatment-Related Adverse Effects in Lung Cancer Patients after Stereotactic Ablative Radiation Therapy

**DOI:** 10.1155/2018/6483626

**Published:** 2018-10-04

**Authors:** Stamati Morias, Loredana G. Marcu, Michala Short, Eileen Giles, Andrew Potter, Justin Shepherd, Thanh Gierlach, Eva Bezak

**Affiliations:** ^1^Cancer Research Institute and School of Health Sciences, University of South Australia, Adelaide SA 5001, Australia; ^2^Faculty of Science, University of Oradea, Oradea 410087, Romania; ^3^Genesis Care, Adelaide, South Australia 5005, Australia; ^4^Radiation Oncology, Royal Adelaide Hospital, Adelaide, South Australia 5000, Australia; ^5^Department of Physics, University of Adelaide, North Terrace, Adelaide, South Australia 5005, Australia

## Abstract

**Introduction:**

Lung cancer is a disease which, despite the advancements in treatment, still has a very poor 5-year survival rate. Stereotactic ablative radiation therapy (SABR) is a highly advanced, sophisticated, and safe treatment which allows patients with early stage lung cancer to be treated effectively without invasive procedures and with excellent clinical outcomes. Avoiding surgery minimises morbidity and recovery time, bettering patients' quality of life. Furthermore, SABR allows patients unsuitable for surgery to still undergo curative treatment.

**Methods:**

We aimed to review SABR-related normal tissue toxicities reported in the literature. While many studies assess safety, clinical efficacy, and disease control of SABR for lung cancer, the number of comprehensive reviews that analyse SABR-related side-effects is scarce. This integrative review summarises the toxicities reported in literature based on published clinical trials and tumour location (central or peripheral tumours) for available SABR techniques. Given that the majority of the clinical studies did not report on the statistical significance (e.g.,* p*-values and confidence intervals) of the toxicities experienced by patients, statistical analyses cannot be performed. As a result, adverse events are compiled from clinical reports; however, due to various techniques and nonstandard toxicity reports, no meta-analysis is possible at the current stage of reported data.

**Results:**

When comparing lobectomy and SABR in phase III trials, surgery resulted in increased procedure-related morbidity. In phase II trials, very few studies showed high grade toxicities/fatalities as a result of SABR for lung cancer. Gross target volume size was a significant predictor of toxicity. An ipsilateral mean lung dose larger than 9 Gy was significantly associated with radiation pneumonitis.

**Conclusions:**

Based on the studies reviewed SABR is a safe treatment technique for lung cancer; however, further well-designed phase III randomised clinical trials are required to produce timely conclusive results and to enable their comparison and statistical analysis.

## 1. Introduction

Lung cancer is the leading cause of cancer-related death in both males and females worldwide, with approximately 1.59 million deaths each year (19.4% of total cancer-related deaths) [1]. Treatment options for lung cancer include surgery (thoracotomy with lobectomy or wedge resection), radiation therapy and chemotherapy, and often a combination of these modalities. Patients with early stage resectable lung cancer undergo surgery, while those with unresectable disease receive radiation therapy in combination with chemotherapy. Radiation therapy is the standard of care for 70 - 90% of patients with localised small cell lung cancer (SCLC) and 60 - 70% of patients with advanced disease, while approximately 19% of patients with early stage of non-small-cell lung cancer (NSCLC) receive radiotherapy as definitive treatment [2]. This increases to 52% of patients with late stage disease.

Several techniques have been designed and implemented over the years to deliver highly conformal radiation therapy: three-dimensional conformal radiation therapy (3DCRT), intensity modulated radiation therapy (IMRT), volumetric modulated arc therapy (VMAT), and proton beam therapy (PBT). Stereotactic ablative radiation therapy (SABR) is a hypofractionated dose regimen whereby relatively small tumours are ablated using stereotactic precision, which can be delivered via any of the aforementioned techniques. SABR has greatly developed over the last two decades through advancements in treatment delivery conformity, treatment techniques and immobilisation [3–7].

## 2. SABR-Related Normal Tissue Toxicity

To minimise the risk of treatment toxicities, dose-volume guidelines are set at treatment planning to ensure that tolerance doses of tissues such as the contralateral lung, heart, spinal cord, chest wall, ribs and skin are not exceeded. The lung dose-volume guidelines in SABR are different to those used in conventional radiation therapy mostly because of the large difference in dose/fractionation used for SABR treatments [8]. In SABR for lung cancer, the high-dose volumes are typically small and dose gradients are very steep [8]. Due to the multiple beams and angles used in SABR, a larger volume of lung is spared excessive radiation dose [8]. If dose-volume specifications are not respected at the treatment planning stage, patients will be at risk of developing a range of radiation-induced toxicities. These include radiation lung injury (discussed below), oesophagitis, radiation dermatitis, chest wall pain/thoracic pain, rib fracture, bronchial stricture, pleural effusion and brachial plexopathy [9].

For centrally located tumours, there is a higher risk of radiation injury as tumours are closer to the bronchial tree and other important mediastinal structures [9]. Rare cases of toxicities resulting from central SABR are oesophageal fistula and bronchial stenosis resulting in atelectasis or bronchial necrosis [9].

Radiation lung injury is generally categorised into two phases: (a) early, for example radiation pneumonitis, which typically occurs within the first three months post-SABR and (b) late, for example radiation-induced pulmonary fibrosis, which can occur six months to several years post-SABR [30]. Symptoms of radiation-induced lung injury can include cough and dyspnoea, with the more extreme lung fibrosis requiring oxygenation and assisted ventilation [9].

According to a review conducted by Ricardi et al. (2015), most of the radiation pneumonitis experienced by SABR patients is RTOG grade 1 or 2 and mostly asymptomatic, with less than 8% of patients experiencing grade 2 or greater radiation pneumonitis requiring intervention [6]. The low rates of pneumonitis in SABR as compared to conventional radiotherapy are likely due to the parallel architecture of the lung, which is more sensitive to high volumes of low doses (conventional radiotherapy) than to high doses delivered to smaller volumes (SABR). Radiation-induced lung fibrosis can appear six months to one year post-SABR [31] and is characterised by the proliferation of fibroblasts and myofibroblasts which deposit connective tissue such as collagen, and extracellular matrix into the pulmonary tissue, causing the alveoli to collapse [30, 31]. This comes as a result of cytokines and other cellular components migrating to the site of irradiation and causing a continual inflammatory process [30] which can lead to respiratory distress and right-sided heart failure [30, 32].

Oesophageal toxicity can also occur from pulmonary SABR. Radiation oesophagitis can range from mild inflammation to oesophageal stricture, perforation or a fistula [9]. The presence of a fistula or a perforation in the oesophagus can result in fatal haemoptysis or sepsis.

Chest wall pain may be a side-effect experienced during or post-SABR. It is more common in patients whose tumours are in peripheral lung, thus closer to the chest wall. While the exact mechanism of chest wall pain is poorly understood, the intercostal nerves have been linked to this side-effect [9].

Brachial plexopathy is more commonly seen in patients with apically situated lung tumours [9]. The mechanism of brachial plexopathy is believed to be linked to loss of myelin sheath, which may result in upper limb paralysis and neuropathic pain [9]. Oesophageal toxicity and brachial plexopathy are likely a function of the anatomic location of the tumour: peripheral tumours are more likely to have treatment fields near plexus, while fields for central tumours are more likely to encompass areas of the oesophagus.

## 3. Aim of the Review

The aim of this review was to evaluate SABR-related normal tissue toxicities reported in the literature. While many studies assess safety, clinical efficacy, and disease control of SABR for lung cancer, the number of comprehensive reviews that analyse SABR-related side-effects is scarce. This integrative review summarises the toxicities reported in the literature based on published clinical trials, tumour location (central or peripheral tumours) for available SABR techniques. Given that the majority of the clinical studies did not report on the statistical significance (e.g.,* p*-values and confidence intervals) of the toxicities experienced by patients, statistical analyses cannot be performed.

As a result,

(a) adverse events are compiled from clinical reports; however, due to various techniques and nonstandard toxicity reports, no meta-analysis is possible at the current stage of reported data;

(b) the extracted data is tabulated to assist the reader in evaluating adverse events related to each SABR technique and in identifying advantages/disadvantages of each.

## 4. Methods

A literature search was conducted on Medline to identify literature fulfilling the aim of this review. The final search was conducted in January 2017 and was designed to identify clinical trials from the year 2000 onwards in English only. The search strategy is shown in Table 1. A total of 846 papers remained after the search was limited to SABR, toxicities, adverse events and normal tissue complication, excluding stereotactic radiosurgery and was further narrowed to 432 papers when only lung papers were included and to 88 papers when limited to clinical reports, ranging from case studies to phase III clinical trials. These 88 papers were screened by four authors (SM, MS, EG, EB) based on titles and abstracts to remove irrelevant papers yielding 71 articles. After quality check of the manuscripts identified, those that did not specifically discuss clinical SABR results (e.g., physics studies) were removed. The final article count was 66.

## 5. Results

### 5.1. SABR Toxicity Based on Clinical Trial Results

Of the 66 clinical studies obtained from Medline, 22 were phase I-III clinical trials: 4 phase I trials, 16 phase II trials and 2 phase III trials (Table 2). The majority of the remaining papers (67%) were retrospective studies. Three review papers were also identified.

To date, the only phase III randomised clinical trials involving SABR-related toxicity are the STARS and ROSEL trials that were designed to compare SABR to surgical resection for stage I NSCLC [10, 33, 34]. Patients were randomised to either surgery or SABR. Patients in the STARS trial with peripherally located tumours were treated with 54 Gy in three fractions, and those with central tumours were treated with 50 Gy in four fractions [10]. Patients in the ROSEL trial were treated with either 54 Gy in three fractions or 60 Gy in five fractions [10]. Both phase III trials were terminated due to lack of recruitment (58 patients) [33, 34]. The results from the trials, however, were synthesised into a pooled analysis by Chang et al. (2015). The results revealed that when comparing lobectomy and SABR, surgery resulted in increased procedure-related morbidity and mortality [10, 35]. The most common surgery-associated morbidities included postoperative pneumonia, atrial fibrillation, myocardial infarction, pulmonary embolism and deep venous thrombosis [10]. Compared to a 0.7% cumulative procedure-related mortality reported after SABR, surgical techniques lead to 5.4% mortality rates within 90 days post-surgery.

The results of phase II trials show that generally, SABR is well tolerated with manageable adverse events in the majority of cases. There were some exceptions, as reported by Timmerman et al. [23] who concluded that SABR may have contributed to grade 5 toxicity. According to their report, 4 deaths occurred due to bacterial pneumonia, 1 patient died of a pericardial effusion and 1 patient experienced fatal massive haemoptysis. It is to be mentioned that this report originates from the early days of SABR, and much effort has been made since to make SABR safer in this patient group. Therefore, the other phase II trials presented in detail in Table 2 showed better toxicity-related results. Grills et al. [19] observed no grade 4 or 5 toxicities, Bral et al. [16] reported 74% of patients with lung toxicity-free survival at 2 years, there were no major adverse events in Collen's cohort [14], Koto et al. [22] observed no ≥ grade 2 toxicity outside the lungs, and in Baumann's trial [20] 35% patients had no pulmonary side-effects from SABR. There was no incidence of SABR-related deaths reported in any of the aforementioned trials.

Toxicities reported within the four phase I clinical trials included radiation pneumonitis, radiation dermatitis, pericardial effusion, tracheal necrosis, hypoxia and bronchitis [26–29]. A multi-institutional phase I study by Onimaru et al. [26] found that the risk of grade 2 radiation pneumonitis at 55 Gy in 4 fractions was above 25%. The statistical significance of this result or any other phase I trial outcome, however, was not provided.

Four of the 66 clinical studies analysed had patient sample sizes of over 400 patients. The studies described toxicities, such as radiation pneumonitis, chronic myositis and radiation-induced dermatitis occurring post-SABR [36–39]. One clinical trial, with 500 patients, showed that on univariate analyses chest wall pain and/or rib fracture was more prevalent in patients with a smaller tumour-chest distance, larger tumour diameter and larger PTV (*p*<0.01) [36].

The prevalence of higher grade toxicities was reported within a large collaborative analysis comprising 483 patients (52% male) [37, 38]. Radiation pneumonitis of grade 2 or higher was experienced by 7% of patients [37, 38]. The median PTV dose delivered in the study was 54 Gy in three fractions [37, 38]. Details on PTV size and location, however, were not available for analysis along with statistical information on the prevalence of radiation pneumonitis. Guckenberger et al. [38] found a correlation between pretreatment pulmonary function and changes to pulmonary function in the long-term for FEV1 (forced expiratory volume) (*p*=0.001), FEV1% (*p*<0.001) and DLCO (diffusion capacity) (*p*=0.02). This study did not specify any variables which remained statistically significant after multivariate analysis and the results were not supported by any of the studies with similar patient cohort sizes. Additionally, the study did not comment on what the specific clinical impact was, as a result to changes to pulmonary function post-SABR.

While all studies were investigating SABR in patients with early stage NSCLC [36–39], the definition of early stage varies according to the staging criteria utilised and the changes in staging system over time (TNM/AJCC) and this was not reported.

### 5.2. SABR Toxicity and Correlation with Tumour Location (Central and/or Peripheral Tumours)

A total of 30 studies of the 66 extracted from Medline, discussed SABR for centrally located tumours (defined commonly within 2cm of the mediastinum or bronchial tree) and peripherally located tumours or a combination of both. Three studies looked solely at central NSCLC tumours (one of which evaluated both centrally located NSCLC and lung metastases), 11 studies examined peripherally located NSCLC tumours or lung metastases and 16 studies looked at both central and peripheral tumours.

Of the three studies which examined SABR for***centrally located NSCLC*** and lung metastases, the sample sizes ranged from 27 to 111 patients, and doses varied from 37.5 Gy in three fractions to 66 Gy in three fractions. A range of toxicities were reported including acute fatigue, acute cough, acute oesophagitis, radiation pneumonitis, dermatitis, musculoskeletal discomfort, pneumonia, pleural effusion, apnoea, brachial plexopathy, partial arm paralysis and skin reactions [23, 40, 41]. In patients experiencing brachial plexopathy, it was found that a significant volume of the brachial plexus received 40 Gy [23, 40, 41]. Timmerman et al. [23] found on both univariate and multivariate analysis that among patients with grade 3 to 5 toxicities, tumour location (hilar/pericentral versus peripheral) was a strong predictor of toxicity (*p*=0.004).

Of the 30 studies, there were 11 which reported SABR treatment for***peripherally located lung tumours***, with most labelled as NSCLC or pulmonary metastases. The sample sizes among the trials varied, ranging from 15 to 127 patients. Total dose and the number of fractions also varied: 66 Gy in three fractions, 60 Gy in three fractions, 48 Gy in four fractions, 60 Gy in five fractions, 57 Gy in three fractions and 45 Gy in three fractions. The toxicities reported included atelectasis, exacerbation of existing pulmonary comorbidity, decrease in pulmonary function tests, cough, dyspnoea, exudate, fatigue, radiation lung fibrosis, radiation pneumonitis, pain, pericardial effusion, rib fracture, hypoxia, brachial plexopathy and skin reactions [11, 13, 24, 26–28, 42–46].

A phase II trial by Chang et al. [15], treating 130 stage one NSCLC patients with 6 MV and 50 Gy in four fractions, observed no differences in the rate of radiation pneumonitis between peripheral and central lesions. This result differs from findings by Timmerman et al. [23], who reported a relationship between toxicity and tumour position. The study also found, that an ipsilateral mean lung dose greater than or equal to 9.14 Gy was significantly associated with radiation pneumonitis on multivariate analysis (*p*=0.005) [15].

A study conducted by Jain et al. [45], with 54 stage 1 NSCLC or single pulmonary metastasis patients, treated patients with 48 Gy in four fractions for NSCLC up to 3 cm in diameter or 52 Gy in four fractions for NSCLC greater than 3cm and for lung metastases. The study established two groups of 27 patients: one group treating the patients over 11 days and the other group treating the patients over 4 days and found that at baseline, dyspnoea was higher in the 11 day group compared with the 4 day group (44.4 versus 25.9,* p*=0.02), along with worsening in role functioning, fatigue and cough in the 11 day group [45]. At one and four months post-SABR, however, there were significantly more patients presenting with an increase in dyspnoea in the 4 day group compared to the 11 day group (44.4% versus 15.4%,* p*=0.02; 38.5% versus 12.0%,* p*=0.03). Furthermore, at four months post-SABR, more patients in the 4 day group had physically worsened compared to those in the 11 day group [45].

Another 16 studies investigated SABR in treating* both central and peripheral lung lesions*. The studies varied greatly in sample size, ranging from 30 patients to 566 patients, with one study not specifying the sample size. A variety of toxicities were presented in the studies and the acute toxicities included cough, chest pain, dyspnoea, radiation pneumonitis and generalised pain, while the chronic toxicities included fatal haemoptysis, rib fracture, cough, radiation pneumonitis, brachial plexopathy, telangiectasia and pain [12, 16, 21, 23, 39, 47–50].

A large multicentre clinical trial by Schanne et al. [39], treated 90 patients for central stage I NSCLC and 476 stage I peripheral NSCLC patients. The median dose for both central and peripheral tumours was 37.5 Gy, administered in 5 fractions for the centrally located tumours and in 3 fractions for the peripheral ones. This study showed very similar results in the occurrence of radiation pneumonitis with 90% of central patients and 94% of peripheral patients experiencing grade two or lower radiation pneumonitis [39]. 9% of central patients and 5% of peripheral patients experienced grade two radiation pneumonitis. No patients experienced grade four radiation pneumonitis in both groups, however, 1% of central patients and less than 1% of peripheral patients died from radiation pneumonitis [39]. With the outcome being very similar, and a lack of statistical information provided, it is difficult to deduce any trends in the occurrence of radiation pneumonitis between patients with central and peripheral stage I NSCLC tumours. There was no data reported on the relationship between radiation pneumonitis and other factors such as age, performance status, gender, baseline respiratory function, preexisting comorbidities, total dose and number of fractions and time period over which SABR was delivered.

A prospective phase II clinical trial by Fakiris et al. [21] with 70 stage I and II NSCLC patients with central and peripheral tumours, treated with 60 Gy in 3 fractions or 66 Gy in 3 fractions, found that while toxicity rates in central tumour patients (27.3%) were higher than the rates of toxicity in peripheral tumour patients (10.4%), the result did not reach statistical significance. This is a similar outcome to that in the multicentre trial by Schanne et al. [39] where the toxicities between central and peripheral tumours were very similar and hard to separate.

### 5.3. SABR Toxicity and Treatment Volume

Among the phase II clinical trials, a study by Lindberg et al. [11], treated 57 patients for stage I and II inoperable NSCLC with 45 Gy in 3 fractions and 6 MV photons with an Elekta linear accelerator, and found that patients with grade 2 or higher radiation-induced fibrosis had larger PTVs compared to patients without fibrosis, but no statistically significant association between fibrosis and lung dose was observed.

Stone et al. [13] investigated the association between SABR delivered in 48 Gy in 4 fractions or 60 Gy in 5 fractions, and the pulmonary function tests. It was found that there was a correlation between gross tumour volume and total lung capacity reduction at 12 months post-SABR. The study also reported that decline in pulmonary function became more apparent with time post-SABR [46]. With regard to toxicities such as radiation-induced lung fibrosis, a multi-institutional study conducted by Lindberg et al. [11] was unable to establish a relationship between total lung dose and lung fibrosis of grade two or higher. Similarly, in their phase II clinical trial of 57 stage I peripheral NSCLC patients treated with 66 Gy in three fractions, Baumann et al. (2008) failed to demonstrate a relationship between the risk of radiation pneumonitis or radiation lung fibrosis to either irradiated lung volumes / lung doses or T1/T2 stage [42].

Stephans et al. [51] reported on the outcome of 45 patients treated with 60 Gy in three fractions. The group investigated post-SABR chest wall toxicity and found that the largest single tumour dimension and PTV were correlated with toxicity (*p*=0.047 and* p*=0.040, respectively). The distance from tumour edge to chest wall and GTV did not reach statistical significance (*p*=0.16 and* p*=0.12, respectively) [51]. This result differs from the outcome reported by Bongers et al. whereby, on multivariate analysis, patients with chest wall pain had larger treatment volumes and had significant chest wall volumes receiving doses between 30 to 50 Gy [36]. On univariate analysis, a significant difference was shown in patients that were younger, had a shorter tumour-to-chest wall distance, larger tumour diameter and larger treatment volume with regard to the presence of chest wall pain versus no chest wall pain (*p*<0.01) [36]. These findings were not confirmed by the other studies with histologically confirmed NSCLC patients – the closest confirmation was by a phase II clinical trial which found that GTV size was a significant predictor of toxicity (in general), on multivariate analysis [23].

### 5.4. SABR Toxicity and Histopathological Confirmation of Disease

Of the 66 clinical trials analysed, 16 trials had histological confirmation of lung cancer. The sample sizes across all 16 studies with histological NSCLC confirmation ranged from 10 to 500. One of the largest studies, which histologically or cytologically confirmed NSCLC, was a multi-institutional Italian clinical trial which had 196 stage I NSCLC patients [49]. All five Italian centres in this multi-institutional study applied homogenous patient inclusion and exclusion criteria. The prescribed total dose ranged from 48 to 60 Gy in 3 to 8 fractions delivered with either 3DCRT, IMRT or VMAT SABR techniques [49]. This study found that toxicities greater than grade 2 were present in 3% of the patient cohort [49]. This study documented one acute toxicity - pulmonary toxicity, with specific information on the type of pulmonary toxicity lacking, and late toxicities which included chest wall pain, neuropathic pain, brachial plexopathy, telangiectasia and rib fractures.* p*-values and confidence intervals on the acute and late toxicities and their relationships with other factors were not provided.

The one multi-institutional study which did not histologically confirm the type of lung cancer in any of the 34 patients (reason for this not stated) appeared to have more patients experiencing acute and late radiation-induced toxicities in comparison to those studies which did have histological confirmation for all patients [52]. The study also mentioned that patients with preexisting comorbidities may have an increased risk of attaining radiation pneumonitis as a result of undergoing SABR which may, in turn, increase the risk of mortality [52].

### 5.5. SABR Toxicity in Operable versus Inoperable Tumours

Nagata et al. [53] investigated the toxicity differences between operable and inoperable stage I NSCLC patients. A total of 164 patients were treated with 48 Gy in 4 fractions, across 15 institutions in this Japanese clinical trial (JCOG0403). It was found that operability did not predict the statistical significance of toxicity rates. No treatment-related deaths occurred in either patient group [53].

Nevertheless, as shown by the outcome of phase III trials that compared SABR-related toxicity with postresection side-effects, SABR was found to have fewer serious treatment-related complications compared to surgery. Therefore based on its toxicity profile SABR may be a favourable treatment approach for operable patients [10].

Also related to surgically resectable tumours, the recently reported interim results of a phase II clinical trial that employed a novel multimodality approach has demonstrated the value of neoadjuvant SABR prior to surgical resection for early stage NSCLC [54]. The MISSILE-NSCLC study included 10 patients treated with presurgery SABR. Overall, the treatment was well tolerated, as out of the 24 toxicities reported, only 3 were grade three or greater, and all in the same patient. While the number of patients accrued by this trial was small, the results of neoadjuvant SABR in patients with resectable tumours warrant further investigations.

## 6. Summary and Conclusions

The systematic literature search found 66 potential sources documenting toxicity post-SABR for lung cancer. Across the studies analysed, a range of acute and chronic toxicities were identified. These toxicities included radiation pneumonitis, radiation-induced lung fibrosis, dyspnoea, cough, chest wall pain, rib fracture, brachial plexopathy, atelectasis, telangiectasia, oesophagitis, fatigue, pleural effusion, pericardial effusion, and a general decrease in pulmonary function. Very few studies showed high grade toxicities/fatalities as a result of SABR for lung cancer. It can, therefore, be concluded that SABR is a safe treatment technique for lung cancer.

The majority of the studies did not provide histological or cytological confirmation of disease or had few patients within a cohort with histological/cytological confirmation of cancer. While patients that are contraindicated to surgery or invasive procedures may not be able to undergo a biopsy due to health concerns, pathological confirmation of disease would ideally allow radiation oncology professionals to specifically tailor the SABR treatment to the patient. Only one multicentre clinical trial, conducted by Ricardi et al. [49], employed consistent patient selection criteria across the centres in the trial, with patients having their histology/cytology status confirmed as NSCLC. There is, also, the potential in retrospective studies analysing prospectively collected data to underreport results. It is vital in these situations for centres to have analogous patient selection criteria, dose and fractionation schemes, toxicity scoring criteria, and the same follow-up period to allow meaningful comparison of results.

Most studies investigated SABR for NSCLC or metastatic lung lesions. This focus can be both positive and negative: while it allows for the studies on NSCLC and lung metastases to be compared with other studies investigating SABR for the same pathology, it does not allow at this stage for accurate and reliable conclusions to be made when comparing studies investigating SABR for NSCLC and lung metastases together. Such analysis might still be useful (subject to data availability), as outcomes of SABR for radioresistant tumour metastases might be different to primary NSCLC. While disease-related outcomes may be quite different when comparing SABR for NSCLC with lung metastases from other primary cancers, toxicity comparisons for the SABR techniques used may be valid. Additionally, the stage of the lung cancer influences the patient's performance status, along with other patient comorbidities, making comparison between NSCLC and lung metastases toxicities difficult. Within the dataset, there were studies that failed to mention the pathology of the lung cancers being treated.

There was also a strong trend towards studies investigating SABR for peripheral lung lesions, with, what appeared to be, studies avoiding SABR for central lung cancer lesions. Whether this is to avoid the risks and potential downfalls to patients when treating central lesions or to solely investigate peripheral lesions, more clinical trials examining the effect of SABR for centrally located lung cancer are essential to develop a more sophisticated understanding of the toxicities that are inherent in SABR.

Additionally, it would be valuable to investigate SABR for lung cancer for males and females and report on toxicities separately. This would allow one to determine whether there is a correlation between gender, and any other gender-specific factors, and the incidence of toxicity.

The clinical trial sample sizes varied greatly, ranging from as high at 566 to a low of nine patients. In this situation, it is difficult to compare the results and statistical parameters from the studies, where provided. Furthermore, not all reported doses and fractionations are unequivocal as the amount of PTV coverage, prescription isodose line, and heterogeneity corrections likely varied between studies.

Studies in general lacked a comprehensive toxicity analysis after SABR. Instead, most studies focused on commonly occurring toxicities such as chest wall pain and radiation pneumonitis. Comparison between the studies that focus solely on a particular toxicity is possible, but does not enable one to investigate the other toxicities that occurred during that clinical trial, while the toxicity under observation occurred. Other questions arise as several studies claimed that some toxicities, such as pneumonia and bronchitis, were radiation-induced. While they may be radiation-related, the toxicities could also be nosocomially acquired or community acquired, as opposed to SABR-related. Compounding the difficulty in comparing the toxicities reported were the range of toxicity grading criteria employed.

Some of the conclusions derived from the clinical trials can be summarised as follows.

*Phase III Trials*

- When comparing lobectomy and SABR, surgery resulted in an increase in procedure-related morbidity.

*Phase II Trials*

- Very few studies showed high grade toxicities/fatalities as a result of SABR for lung cancer.
- Patients with grade 2 or higher radiation-induced fibrosis had larger PTVs compared to patients without fibrosis, but no association between fibrosis and lung dose was observed.
- GTV size was a significant predictor of toxicity.
- Chest wall pain and/or rib fracture was more prevalent in patients with a smaller tumour-chest distance, larger tumour diameters, and larger PTV.
- An ipsilateral mean lung dose larger than 9 Gy was significantly associated with radiation pneumonitis.
- While some trials found no difference in the occurrence of radiation pneumonitis between peripheral and central lesions, others reported a relationship between toxicity (grade 3 to 5) and tumour position (hilar/pericentral versus peripheral).

The results presented in this review support the case for further investigation. Based on the studies reviewed SABR is a safe treatment technique for lung cancer; however, more research needs to be conducted into the area of toxicities, both acute and chronic in nature, for more conclusive results.

## Figures and Tables

**Table 1:** Search strategy.

	**Search terms**	**Results**
**1**	Stereo*∗* body*∗*	2883

**2**	Stereo*∗* ablat*∗*	562

**3**	1 or 2	3348

**4**	SABR or SBRT	2633

**5**	3 or 4	3623

**6**	Stereo*∗* radiosurg*∗* or SRS or neurosurg*∗*	82236

**7**	5 NOT 6	3419

**8**	Side effect*∗*	253907

**9**	Adverse effect*∗*	135864

**10**	8 or 9	377660

**11**	Norm*∗* tissue compl*∗* or NTCP	1992

**12**	Toxic*∗*	631528

**13**	10 or 11 or 12	961736

**14**	7 and 13	1155

**15**	**Limit 14 to English language, humans, year 2000 onwards, all clinical trials, journal articles and reviews**	846

**16**	Limit 15 to clinical study or clinical trial, all or clinical trial, phase I or clinical trial, phase II or clinical trial, phase III or clinical trial, phase IV or clinical trial or controlled clinical trial or meta-analysis or multicentre study and cancer	197

**17**	15 and lung*∗*	432

**18**	15 and prostate	132

**19**	15 and liver	151

**20**	Limit 19 to clinical study or clinical trial, all or clinical trial, phase I or clinical trial, phase II or clinical trial, phase III or clinical trial, phase IV or clinical trial or comparative study or meta-analysis or multicentre study	45

**21**	Limit 17 to (case reports or clinical study or clinical trial, all)	71

**22**	21 NOT physic*∗*	87

**23**	20 NOT physic*∗*	45

**Table 2:** SABR-related toxicities in clinical trials.

**Author**	**Sample size**	**Treatment Details**	**Toxicities**	**Comments**
**PHASE 3 CLINICAL TRIALS**				

Chang et al. [10] Pooled analysis of 2 independent randomised phase 3 trials 2015	58 patients with cT1-2aN0M0 operable NSCLC	STARS trial - 54Gy/3# [peripheral] 50Gy/4# [central] ROSEL trial - 54Gy/3# or 60Gy/5# STARS - CyberKnife ROSEL - linear accelerator.	3 patients had grade 2 SABR-related events - 3 had CW pain, 2 had dyspnoea and 1 had fatigue and a rib fracture. No patients experienced grade 4 or 5 events.	Toxicity scored with NCI CTCAE v.3.0. This study compared lobectomy and SABR - found that surgery results in increase procedure-related mortality and morbidity compared with SABR.

**PHASE 2 CLINICAL TRIALS**				

Lindberg et al. [11] Analysis of phase 2 multi-centre trial results 2015	57 patients with T1-2N0M0 inoperable NSCLC Male n=26; Female n=31	45Gy/3# prescribed to 67% isodose line encompassing PTV. Centre of PTV receiving 66Gy/3# Elekta linear accelerator. 6MV.	No grade 5 toxicities. 17 grade 3-4 toxicities, 3 presenting > 36 months - rib fracture, dyspnoea, ventricular tachycardia. Within first 36 mths - 1 patient with grade 4 dyspnoea died of respiratory failure not attributed to SABR. Late toxicities - 3 grade 3 1 patient grade 3 rib fracture. 1 patient with grade 3 dyspnoea. 1 patient with ventricular tachycardia. Atelectasis: Early - grade 2 [2] COPD exacerbation: Late - grade2 [2]. Cough - Early - grade 2 [5], grade 3-4 [1] - Late - grade 2 [1] Dyspnoea - Early - grade 2 [9], grade3-4 [3] - Late - grade 2 [2], grade 3-4 [1] Exudate - Early - grade 2 [3], grade 3-4 [1] - Late grade 2 [1]. Fatigue - Early - grade 2 [8], grade 3-4 [1]. Fibrosis - Early - grade 2 [9], grade 3-4 [3] Heart - Early - grade 2 [1], grade 3-4 [1]. Lung infection - Early - grade 2 [1], grade 3-4 [1] - Late grade 2 [1]. Pain - Early - grade 2 [2], grade 3-4 [2] Pericardial effusion - Early - grade 2 [1] Pneumonitis - Early - grade 2 [6]. Rib fracture - Early - grade 2 [2] - Late grade 2 [5]. grade 3-4 [1] Skin and subcutaneous - Early - grade 2 [5]	Toxicity scored with NCI CTCAE v.4.0 and the RTOG late toxicity scale. Patients with ≥grade 2 fibrosis had larger PTVs compared to patients without fibrosis but no association between dose to lung and fibrosis was observed. For patients with ≥grade 2 fibrosis, no association between lung dose and fibrosis was observed.

Nuyttens et al. [12] Phase 2 clinical trial 2015	30 patients with lung oligometastases Male n=16; Female n=14	Large peripheral [>3cm] - 60Gy/3#; Small peripheral [≤3cm] - 30Gy/1#; Central tumours - 60Gy/5# CyberKnife.	3 patients acute grade 3 fatigue - 1 grade 2 and 1 grade 1 fatigue pre-SABR. 2 acute grade 3 chest pain - 1 with grade 2 pain pre-SABR. 4 acute grade 3 dyspnoea - 2 of which had grade 1 dyspnoea and 1 with grade 2 dyspnoea pre-SABR. Chest pain resolved in 3 patients but worsened in 5 patients. 1 patient chronic grade 3 pain, 1 with grade 3 fatigue and 1 with grade 3 RP. 6 chronic grade 2 fatigue, 4 grade 2 cough, 1 grade 2 dyspnoea, and 1 grade 2 chest pain.	Toxicity scored against NCI CTCAE v3.0.

Stone et al. [13] Phase 2 trial 2015	127 patients with Stage 1 NSCLC Male n=53; Female n=74	48Gy/4# or 60Gy/5# Elekta Synergy or Axesse [some IMRT and VMAT]	At 12 months, there was a 4.1% decline in FEV1 and at 24 months a 7.6% decline. At 9 months, there was a 6.1% decline in DLCO and a 5.2% decline at 12 months. At 12 months, there was a 5.7% decrease in FVC and at 24 months a 5.2%. 3.1% grade 2 RP and 0.8% grade 3 RP. No grade 3 FEV1 or FVC toxicities were experienced. 95% experienced grade 0-1 FEV1 decline at 6 months and 92% at 12 months. 97% experienced a grade 0-1 FVC decline at 6 months and 95% experienced a decline at 12 months. 85% experienced grade 0-1 DLCO toxicity at 6 months and 88% experienced at 12 months.	RTOG pulmonary function text toxicity scale and NCI CTCAE v.3.0. used to grade toxicity. The 12 month decline in FEV1 only observed in patients with a baseline FEV1 ≥50% compared to patients with a baseline FEV1 ≤50%. Decline in DLCO at 12 months in both the baseline-corrected DLCO ≥50% and baseline corrected DLCO ≤50%. A correlation between GTV volume and TLC reduction at 12 months - but no significant correlations between pulmonary tests and other volumetric parameters.

Collen et al. [14] Phase 2 clinical trial 2014	26 patients with oligometastatic NSCLC Male n=20; Female n=6	50Gy/10# TomoTherapy Hi-Art II system or VERO SBRT system	4 patients acute grade 2 toxicity - 1 with dyspnoea, 2 with dysphagia and 1 with RP. No major late toxicities were observed. Toxicity rate of 8%.	NCI CTCAE v.3.0. used to score the patient toxicities.

Chang et al. [15] Prospective analysis of phase 2 clinical trial 2012	130 patients with Stage 1 NSCLC Male n=67; Female n=63	50Gy/4# Machine not specified. 6MV used.	15 patients with RP - 9.2% had grade 2 RP and 2.3% had grade 3 RP. 12 patients with RP - 8.5% experienced grade 2 CW pain and 0.8% experienced grade 3 CW pain. 8 patients with dermatitis - 6.2% with grade 2 or 3 dermatitis. 1 patients [1.5%] developed grade 1 oesophagitis.	No difference in occurrence of RP between central and peripheral lesions. Ipsilateral mean lung dose ≥9.14Gy was significantly associated with RP on multivariate analysis, p=0.005. In patients with an ipsilateral mean lung dose <9.14Gy, only 1 patient had grade 2-3 RP [p<0.001]. On univariate analysis, lung volumes from 5-40Gy and mean lung dose were associated with the incidence of grade 2-3 RP, p<0.05.

Bral et al. [16] Prospective phase 2 trial 2011	40 patients with T1-3N0M0 NSCLC Male n=33; Female n=7	60Gy/3# for peripherally located lesions - 60Gy/4# for central lesions Novalis system. Energy not specified.	≥grade 3 late toxicity was seen in 8 patients [20%]. The average decline in FEV1 and DLCO was 3% for both. Grade 1 FEV1 changes occurred in 18% and grade 2 changes occurred in 15%. Grade 1 DLCO changes occurred in 13% and grade 2 changes occurred in 20%. 1 patient with a dermal fibrotic reaction developed an ulcer. 6 patients persistent grade 1 or 2 weight loss and 1 fatigue. 2 patients acute grade 3 RP; 8 chronic grade 1 RP, 5 grade 2 RP, 6 grade 3 RP. 21 acute grade 1 cough, 5 grade 2 cough, 2 grade 3 cough; 3 chronic grade 1 cough, 1 chronic grade 2 cough. 2 acute grade 1 dyspnoea, 2 grade 2 dyspnoea; 3 chronic grade 1 dyspnoea, 3 grade 2 dyspnoea, 1 grade 3 dyspnoea. 1 chronic grade 3 stenosis. Dysphagia, nausea, anorexia, pyrosis, fatigue, skin reactions, fever, pain in several patients.	NCI CTCAE v.3.0. used to score the patient toxicities along with the RTOG acute and late morbidity scoring system. Lung toxicity-free survival estimate at 2 years was 74%. Group analysis showed a correlation between 2 year lung toxicity-free survival and location: peripheral 84% vs. central 60% [p=0.06] and PTV size [<65cc vs. ≥65cc, p=0.02]. No correlation between the degree of lung toxicity and the absolute or relative lung volume receiving 20Gy or 10Gy, mean lung dose, the gradient, CI, or the total lung volume. Baseline FEV1 or DLCO did not predict for lung toxicity. Pulmonary function test changes at 3 months were not related to the lung doses, mean lung dose, the gradient, CI or the total lung volume.

Timmerman et al. [17] RTOG trial 0236 Phase 2 North American multicentre trial 2010	55 patients with T1-2N0M0 NSCLC Male n=21; Female n=34	54Gy/3# Machine not specified. 4-10MV used.	7 grade 3 events – 2 grade 4 events. Blood / bone marrow decrease in 6 patients - 3 grade 1, 1 grade 2, 2 grade 3. CV issues in 2 patients - 1 grade 1, 1 grade 2. Coagulation in 2 patients - 1 grade 1, 1 grade 3. Dermatological issues in 7 patients - 3 grade 1, 2 grade 2, 2 grade 3. GIT issues in 6 patients - 4 grade 1, 1 grade 2, 1 grade 3. Haemorrhage in 2 patients - 2 grade 2. Infection in 3 patients - 1 grade 2, 2 grade 3. Lymphatic issues in 2 patients - 2 grade 1. Metabolic issues in 5 patients - 2 grade 1, 1 grade 2, 1 grade 3, 1 grade 4. Musculoskeletal issues in 11 patients - 3 grade 1, 5 grade 2, 3 grade 3. Neurological pain in 6 patients - 3 grade 1, 2 grade 2, 1 grade 3. Pain in 14 patients - 5 grade 1, 9 grade 2. Upper respiratory 33 patients - 11 grade 1, 13 grade 2, 8 grade 3, 1 grade 4. Renal issues in 1 patient - 1 grade 1. FEV1 decrease - 2 grade 3. Hypocalcaemia - 1 grade 4. Hypoxia - 2 grade 3. Pneumonitis NOS - 2 grade 3. Pulmonary function test decrease NOS - 3 grade 3, 1 grade 4.	Toxicity grading system not specified.

Ricardi et al. [18] Prospective phase 2 trial 2010	62 patients with Stage 1 NSCLC Male n=52; Female n=10	45Gy/3# Elekta Precise linear accelerator. 6-10MV.	10% transient mild erythema. 10% dyspnoea and cough. 7% transient chest pain. 15% fatigue. 2 patients ≥ grade 3 RP. 3 patients radiation-induced fibrosis. 1 patient rib fracture post-SABR. 3 patients [4.8%] chronic thoracic pain syndrome.	Patients toxicities scored using the RTOG acute and late radiation toxicity scoring system.

Grills et al. [19] Prospective phase 2 trial 2010	124 patients with Stage 1 NSCLC 60% of patients were female.	T1 tumours - 48Gy/4#; T2 tumours - 60Gy/5#	No grade 4 or 5 toxicities were experienced. SABR related toxicities- 21% grade 1-3 RP. 9% grade 1, 9% grade 2 and 2% grade 3. 12% grade 1-2 rib fractures with 7% being symptomatic requiring treatment. 27% grade 1 fatigue. 38% grade 1 radiation dermatitis. 21% grade 1-3 acute dyspnoea. 8% developed grade 1-3 chronic dyspnoea. 4% developed acute myositis; 10% developed chronic myositis.	NCI CTCAE v.3.0. used to score toxicities. Study comparing SABR and wedge resection for the stage 1 NSCLC. The occurrence of myositis was related to tumour-chest wall proximity.

Baumann et al. [20] Prospective phase 2 trial 2009	57 patients with T1-2N0M0 inoperable NSCLC Male n=26; Female n=31	66Gy/3# Linear accelerator. 6MV	16 patients [28%] grade 3 toxicity and 1 patient grade 4 dyspnoea. No lethal effects from SABR. 14 patients [35%] had no pulmonary adverse effects from SABR. 26% acute dyspnoea, 23% acute pleural effusion, 11% acute atelectasis, 4% acute pneumonia, 18% acute pneumonitis, 35% acute fibrosis, 4% acute oesophagitis, 19% acute thoracic pain, 30% acute cough, 4% acute heart failure, 2% acute rib fracture, 30% acute fatigue, 44% acute skin reactions. 18% chronic dyspnoea, 9% chronic pleural effusion, 12% chronic atelectasis, 35% chronic fibrosis, 6% chronic thoracic pain, 3% chronic heart failure, 15% chronic rib fractures, 9% chronic fatigue.	NCI CTCAE v.2.0 used to score patient toxicities.

Fakiris et al. [21] Prospective phase 2 trial 2009	70 patients with stage 1 NSCLC	60Gy/3# - T1 tumours; 66Gy/3# - T2 tumours	10.4% of patients with peripheral tumours had grade 3-4 toxicity; 27.3% of patients with central tumours developed grade 3-4 toxicity. 27.7% of the patients not oxygen dependent pre-SABR became oxygen dependent at a median of 55.6 months post-SABR. 6 grade 3, 1 grade 4 and 5 grade 5 toxicities possibly SABR-related. 1 grade 4 apnoea, 1 pneumonia, 2 pleural effusion, 2 with pulmonary function decline, 1 with anxiety. The grade 5 toxicities were - 3 pneumonia, 1 haemoptysis and 1 respiratory failure. 2 grade 3 toxicities - pneumonia and erythema - were attributed to SABR.	Toxicity scored with NCI CTCAE v.2.0. Study states that there was no significant difference between patients with peripheral and central tumours [p=0.697]. While estimate toxicity rates in central tumour patients [27.3%] were near 3 times higher than the rates of peripheral tumour patients [10.4%], this did not reach statistical significance.

Koto et al. [22] Phase 2 clinical trial 2007	31 patients with medically inoperably stage 1 NSCLC Male n=25; Female n=6	45Gy/3#; if close to OAR - 60Gy/8# Varian linear accelerator - Clinac23EX. 6MV	24 patients grade 1 acute RP, 3 patients grade 2 acute RP, and 1 patient experienced grade 3 acute RP. 2 patients did not developed RP. No ≥grade 2 toxicity was observed outside the lungs. 1 patient developed grade 3 pulmonary toxicity due to an upper bronchus obstruction.	Toxicity graded according to NCI CTCAE v.3.0.

Timmerman et al. [23] Phase 2 trial 2006	70 patients with stage 1 NSCLC Male n=34; Female n=36	T1 tumours - 60Gy/3#; T2 tumours - 66Gy/3#	58 of the 70 patients had grade 1-2 toxicity - fatigue, musculoskeletal discomfort and RP. 8 patients were identified as having grade 3-4 toxicity post-SABR: decreased pulmonary function, pneumonia, pleural effusion, apnoea and skin reactions. SABR may have contributed to the death of 6 patients - grade 5 toxicity. 4 deaths were via bacterial pneumonia - 1 patient died of a pericardial effusion and 1 patient experienced fatal massive haemoptysis.	Toxicity graded according to NCI CTCAE v.2.0. In both univariate and multivariate analyses of those patients experiencing grade 3-5 toxicities, the location of the tumour [hilar/pericentral vs. peripheral] was a strong predictor of toxicity, p=0.004. On multivariate analysis, the GTV size was a significant predictor of toxicity.

Paludan et al. [24] Phase 2 trial 2006	28 patients with medically inoperable stage 1 NSCLC Male 14; Female 14	45Gy/3# Siemens Primus or a Varian Clinac 2100/2300. Energy not specified.	Aggravated dyspnoea was registered in 11 patients [40%] of the cohort. 1 of these patients experienced an increase of 1 grade - the remaining 7 experienced an increase of 2 grades.	WHO toxicity scoring of SABR side-effects. No significant association between DVH parameters and changes in dyspnoea. No association between age, gender, tumour volume and localisation performance status, and aggravated dyspnoea. COPD showed the closest association with the occurrence of aggravated dyspnoea post-SABR. No association between dose, irradiated lung volume and dyspnoea.

Okunieff et al. [25] Phase 2 study 2006	49 patients with lung metastases Males n=22; Females n=27	Preferred - 50Gy/5# Novalis Shaped Beam Surgery.	17 patients [35%] grade 1 pulmonary toxicity. 3 patients [6.1%] grade 2 complaints and 1 patient had grade 3 toxicity. One patient grade 3 malignant pleural effusion - this was managed with pleurocentesis and sclerosis. Most grade 2 toxicity was a cough. 4.1% grade 1 oesophagitis, 2% grade 3 pleural effusion, 2% grade 3 pericardial effusion. 35% grade 1 RP/pulmonary infiltrates. 6.1% grade 2 RP/pulmonary infiltrates.	Toxicity graded against the NCI CTCAE v.3.0. Metastases from breast, lung, renal, endocervical and colon primaries. Toxicity was not clearly associated with V20.

**PHASE 1 CLINICAL TRIALS**				

Onimaru et al. [26] Multi-institutional phase 1 trial 2015	15 patients with peripheral stage 1 NSCLC Male n=8; Female n=7	40Gy; 45Gy; 50Gy; 55Gy; 60Gy Machine not specified. 4-6MV.	1 patient grade 2 RP - this patient was treated with 60Gy/4# at D95 of PTV. The remaining patients had grade 1 or lower RP. All patients had RP within 180 days post-SABR. 2 patients developed a cough after 180 days. 4 patients dyspnoea after 180 days. 1 patient hypoxia 180 days post-SABR. 3 patients fractures 180 days post-SABR. 1 patient brachial plexopathy.	Toxicity graded with NCI CTCAE v.3.0. Probability of the percentage of grade 2 RP at 55Gy is above 25%. Recommended dose for SABR T2N0M0 NSCLC with PTV <100cc is 55Gy/4#.

Takeda et al. [27] Phase 1 trial 2014	15 patients with peripheral lung tumours Male n=10; Female n=5	60Gy/5# to 60% isodose line - this equals 100Gy/5#. Varian linear accelerator - Dynamic conformal multiple arc therapy. 6MV.	6 months post-SABR, 5 patients were grade 0 RP, 9 patients were grade 1 RP, 1 patient was grade 2 RP. The usual RP occurred in and around the PTV.	NCI CTCAE v.4.0. used to score toxicities.

Rusthoven et al. [28] Multi-institutional phase 1/2 trial 2009	38 patients with lung metastases	48Gy/3#, 54Gy/3#, 60Gy/3#. Linear accelerator. 6-15MV	No cases of grade 4-5 toxicity. 3 patients grade 3 toxicity. 10.5% grade 2 radiation dermatitis. 2.6% symptomatic grade 2 RP. 2 of 3 cases of grade 3 toxicity involved the chest wall or skin in patients with peripheral lesions.	NCI CTCAE v.3.0. used to score toxicities.

McGarry et al. [29] Phase 1 study 2005	47 patients with stage T1a-b NSCLC Male n=31; Female n=16	24Gy/3# Machine not specified. 6MV photons [less frequently 15MV]	4 patients grade 2-4 RP. 2 patients grade 2-3 pericardial effusion. 1 grade 3 tracheal necrosis. 1 grade 3 dermatitis. 1 grade 3 hypoxia. 1 grade 2 distant pneumonia. 1 patient grade 2 bronchitis.	NCI CTCAE [version not specified] was used to score toxicity. Not specified whether the toxicities are acute or chronic.

## Data Availability

Data (duly referenced) supporting the results reported in this review are presented in a tabulated format and can be found in references provided.
